# LncRNA SNHG3 promotes bladder cancer proliferation and metastasis through miR‐515‐5p/GINS2 axis

**DOI:** 10.1111/jcmm.15564

**Published:** 2020-06-28

**Authors:** Guangcheng Dai, Chenchen Huang, Jinhui Yang, Lu Jin, Kai Fu, Feng Yuan, Jin Zhu, Boxin Xue

**Affiliations:** ^1^ Department of Urology The Second Affiliated Hospital of Soochow University Suzhou China; ^2^ Anhui Medical University Hefei China; ^3^ Department of Andrology Urology Shengli Oilfield Central Hospital Dongying China

**Keywords:** Bladder cancer, GINS2, lncRNA, miR‐515‐5p, SNHG3

## Abstract

Growing evidence suggests that long non‐coding RNAs (lncRNAs) are associated with carcinogenesis. LncRNA small nucleolar RNA host gene 3 (SNHG3) is up‐regulated in various cancers and positively associated with poor prognosis of these cancers. However, the precise role of lncRNA SNHG3 in bladder cancer (Bca) remains unclear. In our research, we first reported that lncRNA SNHG3 was up‐regulated in bladder cancer tissues and positively related to poor clinical prognosis. Moreover, knockdown of lncRNA SNHG3 significantly suppressed the proliferation, migration, invasion and EMT process of Bca cells in vitro and vivo. Mechanistically, we revealed that suppression of SNHG3 evidently enhanced miR‐515‐5p expression and decreased GINS2 expression at posttranscriptional levels. Moreover, SNHG3 positively regulated GINS2 expression by sponging miR‐515‐5p under a competing endogenous RNA (ceRNA) mechanism. To sum up, our study suggested lncRNA SNHG3 acted as a microRNA sponge and an oncogenic role in the progression of bladder cancer.

## 1 INTRODUCTION

Bladder cancer (Bca) is the most prevalent malignant urinary tumour.^1^ Within the increasing in incidence and mortality, bladder cancer has been caused more than 150 000 deaths per annum.^2^ Although clinical therapies have made significant improvements in the past decades, such as surgery, chemotherapy and radiotherapy, the 5‐year survival rate of Bca patients remains at a low level.^3^, ^4^, ^5^, ^6^, ^7^ Lack of early diagnosis and effective treatment may lead to the poor clinical outcome in Bca patients.^8^ Therefore, it is essential to explore novel molecular mechanisms and therapeutic target of bladder cancer.

Long non‐coding RNAs (lncRNAs) are a common type of non‐coding RNAs with 200 nucleotides in length.^9^, ^10^, ^11^ Emerging evidences showed that lncRNAs played vital roles in cancer progression.^9^, ^10^ For example, lncRNA MALAT‐1 is up‐regulated in several cancers, causing poor prognosis of these cancer patients.^12^, ^13^ LncRNA SPRY4‐IT1 could promote the growth and metastasis of Bca via modulating miR‐101‐3p/EZH2 axis.^14^ LncRNA HOXD‐AS1 could sponge miR‐130a to promote the migration and invasion of glioma cells via increasing E2F8 expression.^15^ LncRNA FTX could suppress the proliferation and metastasis of non–small‐cell lung cancer via regulating miR‐200a‐3p/FOXA2 signalling.^16^ Previous study suggested that lncRNA small nucleolar RNA host gene 3 (SNHG3) acted as an oncogene in the progression of various cancers, including hepatocellular carcinoma, colorectal cancer and lung cancer.^17^, ^18^, ^19^ In ovarian cancer, lncRNA SNHG3 is regarded as a regulator of energy metabolism.^20^ In gastric cancer, lncRNA SNHG3 promotes the tumour progression though regulating MED18 methylation.^21^ Although the oncogene roles of SNHG3 were well recognized in a bunch of cancers, the functionality and underlying mechanisms of SNHG3 in Bca remain uncovered.

In this study, we discovered that lncRNA SNHG3 was significantly up‐regulated in 64.3% (45 of 70) of Bca tissues compared with matched adjacent tissues. In addition, the expression of lncRNA SNHG3 was significantly related to tumour size and postoperative metastasis. Moreover, the down‐regulation of lncRNA SNHG3 significantly inhibited the tumorigenesis and epithelial‐mesenchymal transition (EMT) process of bladder cancer cells in vitro and vivo. Mechanistically, we revealed that SNHG3 escalated expression of GINS2 though sponging miR‐515‐5p under the mechanism of ceRNA dependence. Additionally, knockdown of miR‐515‐5p reversed the inhibited tumorigenesis of bladder cancer cells induced by silencing SNHG3. In summary, our study indicated that lncRNA SNHG3 may serve as a novel diagnostic or therapeutic target of bladder cancer.

## 2 MATERIALS AND METHODS

### 2.1 Patients and clinical samples

Between 2013 and 2019, 70 pairs of bladder cancer tissues and adjacent normal tissues were collected from 70 patients during radical cystectomy. Information about the patients and cancer tissues was listed in Table 1. Each patient has previously signed an informed consent. This study has been approved by the ethics committee institution of Soochow University.

**TABLE 1:** Correlation between lncSNHG3 expression and clinicopathological characteristics of 70 patients with bladder cancer

Characteristics	Group	Total	lncSNHG3 expression		*P* value
			High	Low	
Gender	Male	51	37	14	0.115
	Female	19	10	9	
Age (years)	<60	29	11	4	0.587
	≥60	41	36	19	
Tumour size (cm)	<3 cm	37	20	17	0.014*
	≥3 cm	33	27	6	
Histological grade	L	31	23	8	0.263
	H	39	24	15	
T stage	1/2	45	30	15	0.909
	3/4	25	17	8	
Lymph nodes metastasis	Present	8	6	2	0.615
	Absent	62	41	21	
Postoperative metastasis	Metastasis	26	22	4	0.017*
	Non‐metastasis	44	25	19	

* *P* < 0.05 or **P < 0.01 was considered significant (Chi‐square test between 2 groups).

### 2.2 Cell culture

In this study, all the cell lines were obtained from the American Type Culture Collection (ATCC, Manassas, VA, USA). The 5637 cells were cultured in RPMI‐1640 Medium (Invitrogen, Carlsbad, CA, USA). The T24 cells were cultured in the DMEM medium (Invitrogen, Carlsbad, CA, USA). All cultures were supplemented with 10% foetal bovine serum (FBS) and 1% antibiotics. All cell lines were cultured in an incubator at 37°C and 5% CO_2_.

### 2.3 Transfection

Two short hairpin RNAs (shRNAs) targeting lncRNA SNHG3 (shSNHG3‐1 and shSNHG3‐2) and negative control (shCtrl) were purchased from GenePharma (Suzhou, China). The shlncRNA SNHG3‐1 sequence was as follows: 5′‐GGGCACTTCGTAAGGTTTAAA‐3′; the shlncRNA SNHG3‐2 sequence was as follows: 5′‐GGTTGAGTGCAAGATGAGTTA‐3′.^21^ miR‐515‐5p inhibitor (miR inhibitor) and its negative control (miR‐NC) were ordered from RiboBio (Guangzhou, China). All cell transfections were performed by Lipofectamine 3000 (Invitrogen, USA) according to the manufacturer. Plasmids construction and pcDNA3.1‐lncRNA SNHG3 were synthesized by GenePharma (Suzhou, China). For stable transfection, cell lines were all selected for at least 2 weeks with 300 μg/mL of neomycin.

### 2.4 RNA extraction and quantitative real‐time PCR

We used Trizol reagent (Invitrogen, Carlsbad, CA, USA) to extract RNA from tissues or transfected cells. Quantitative real‐time PCR (qRT‐PCR) was performed according to SYBR Green PCR kit (Takara, Dalian, China) and conducted by Roche LightCycler^®^ 480II PCR instrument (Basel, Switzerland). The sequences of the primers used in this research were listed in Table S1. GAPDH or U6 small nuclear RNA was applied as internal control.

### 2.5 Western blotting assay

Protein was extracted by using RIPA reagent (Beyotime, Beijing, China), then isolated by 10% SDS‐PAGE and transferred to PVDF membranes. The membranes were sealed with 5% non‐fat milk for 60 minutes and incubated at 4°C with primary antibodies for 12‐16 hours. Radiograph was monitored though Quantity One software (Bio‐Rad). Antibodies for N‐cadherin, vimentin, E‐cadherin, GINS2 and GAPDH were obtained from Cell Signaling Technology (Danvers, MA, USA).

### 2.6 Cell proliferation assay

We used cell counting Kit‐8 assay (CCK‐8 assay) and colony formation assay to assess cell proliferative ability. For CCK‐8 assay, the absorbance value was calculated at 45nm with microplate reader (Bio‐Rad, Hercules, CA, USA). For colony formation assay, the transfected bladder cancer cells were grown in 6‐well plates at consistency of 1000 cells per well for 2 weeks. The colonies were dyed by crystal violet and swilled by glacial acetic acid, then detected at 595nm by the microplate reader.^22^

### 2.7 Wound healing assay

Wound healing assay was performed to evaluate cell migration activity. Transfected bladder cancer cells were placed in 6‐well plates. When cells grow to 90%‐95% confluence, we scratched wounds though whole layer of cells with the pipette tip. After 24 hours, the images of wound fields were taken by a digital microscope at 10X scene.

### 2.8 Cell invasion assay

A 24 hours after transfection, cells were seeded at density of 1 × 10^5^ cells per well in the upper chamber of transwell insert (8 μm, Corning). Each upper chamber was covered with Matrigel (BD Bioscience). And each of the lower chamber contained 500 μL of medium with 10% FBS. We used 0.1% crystal violet to dye the penetrating cells and observed though a digital microscope at 10X scene. We swilled the stained cells with glacial acetic acid and detected the absorbance value at 595 nm by the microplate reader.

### 2.9 RNA pull‐down assay

T24 and 5637 cells were transfected with biotin‐labelled lncRNA SNHG3 in order to pull down the sponged miRNA. After 12 hours, the pull‐down compounds were handled with RNase‐free DNase I and RNeasy Mini Kit (QIAGEN, Germany). Then, the drawn miRNAs were extracted and quantified by qRT‐PCR. The primers used in this assay and their corresponding sequences are itemized in Table S2.

### 2.10 Nuclear/cytoplasmic fractionation

Nuclear/cytoplasmic fractionation was performed with PARIS Kit (Life Technologies, MA) based on the manufacturer's instructions. After the cell nuclear and cytoplasmic fractionating, we determined the SNHG3 expression with RT‐qPCR and used GAPDH and U6 as internal control.

### 2.11 Dual‐luciferase reporter assay

We constructed lncRNA SNHG3 wild‐type (WT1, WT2) or mutant (Mut1, Mut2) vectors and co‐transfected them, respectively, with miR‐515‐5p mimics or corresponding control (miR‐NC) to bladder cancer cells. The vectors of GINS2 wild‐type (WT1, WT2) or mutant (Mut1, Mut2) were framed and co‐transfected, respectively, with miR‐515‐5p mimics or corresponding control (miR‐NC). After 48 hours, we used Dual‐Luciferase Reporter Assay System (Promega) to measure luciferase activity differences.

### 2.12 Tumour xenografts

The tumour xenotransplantation test has been authorized by the ethics committee institution of Soochow University. Mice were grown in standard laboratory process. Randomly dividing 6 BALB/c‐Nude mice into the shlncRNA SNHG3 group or shCtrl group (3 mice for each group). 5 × 10^6^ 5637 cells were transfected stably with lentivirus wrapped shlncRNA SNHG3 plasmids or control vector, then hypodermically injected under the skin on the back of BALB/c‐Nude mice. The tumours were measured every week since tumour formation, then removed and weighted after 6 weeks.

### 2.13 Statistical analyses

All experiments were repeated at least three times. All data from independent experiments analysed by SPSS 19.0 (SPSS, Chicago, USA) and indicated by mean ± standard deviation (SD). The difference between groups was tested by t test or paired sample *t* test. A *P* value < 0.05 was considered statistically significant.

## 3 RESULTS

### 3.1 LncRNA SNHG3 expressed highly and associated with clinicopathological characteristic in bladder cancer tissues

**FIGURE 1:**
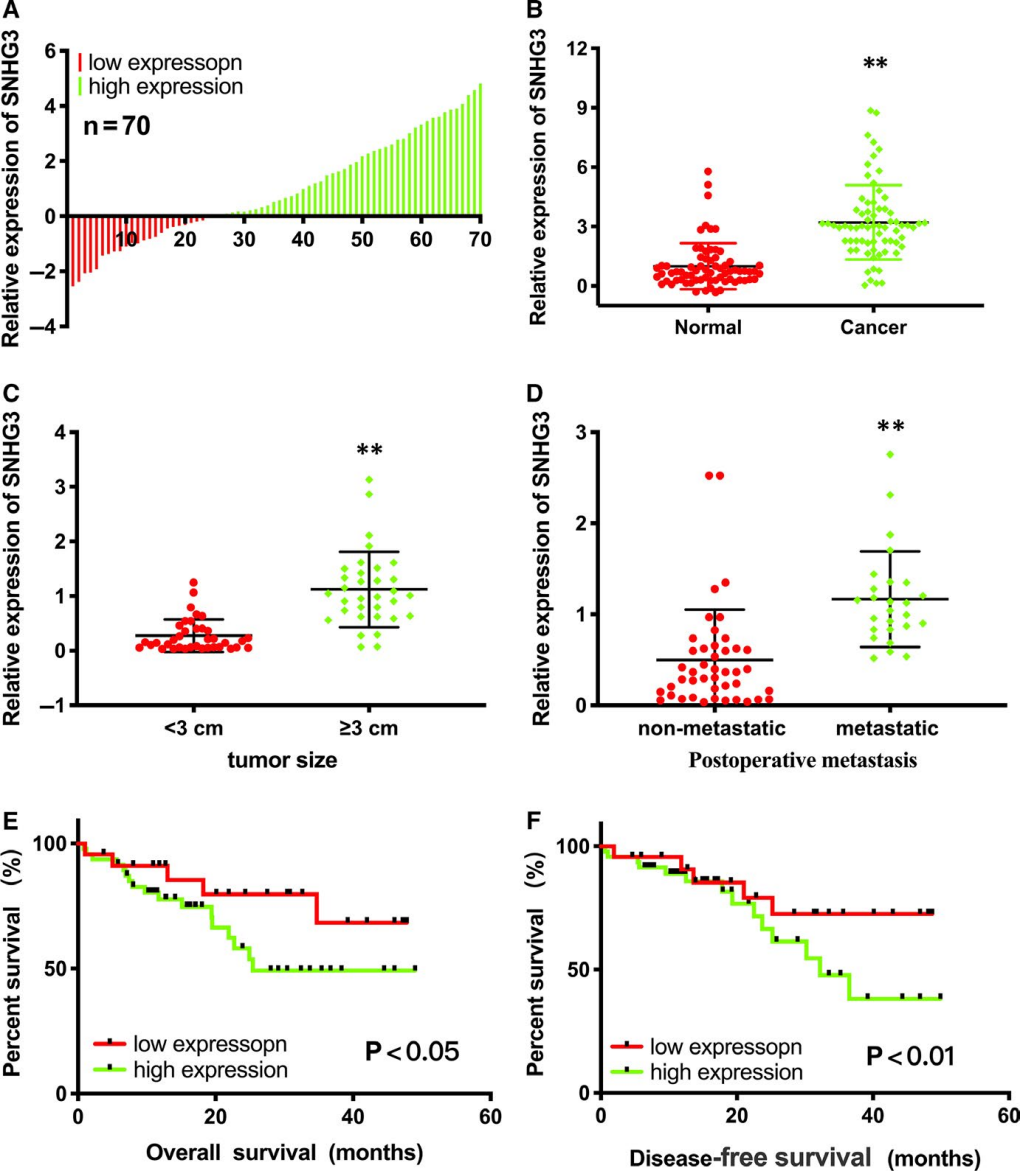
LncRNA SNHG3 expression up‐regulated in 70 bladder cancer tissues and associated with clinicopathological characteristics of patients with bladder cancer. A, Expression level of lncRNA SNHG3 in 70 bladder cancer tissues. B, Expression level of lncRNA SNHG3 was enhanced in 70 bladder cancer tissues compared to adjacent normal tissues. C, lncRNA SNHG3 expression significantly increased in the group with larger tumour size. D, lncRNA SNHG3 expression significantly up‐regulated in the group with postoperative metastasis. E, Higher expression level of lncRNA SNHG3 led to longer overall survival time. F, Higher expression level of lncRNA SNHG3 led to longer disease‐free survival time. Data are represented as mean ± SD. *P < 0.05; **P < 0.01

qRT‐PCR assay was performed to test the lncRNA SNHG3 expression in bladder cancer tissues. Compared to matched normal tissues, the expression of lncRNA SNHG3 was up‐regulated remarkably in 67.14% (47 of 70) of cancer tissues (*P* < .001). These 47 tissues were assigned to the high expression group, and the remaining 23 to the low expression group (Figure 1A). And lncRNA SNHG3 expressed evidently higher in Bca tissues than in corresponding normal tissues (Figure 1B). Moreover, lncRNA SNHG3 expression was positively correlated with tumour size and postoperative metastasis of Bca patients (Table 1). The expression level of lncRNA SNHG3 significantly up‐regulated in the group with tumour diameters over 3 cm (Figure 1C). The expression level of lncRNA SNHG3 significantly increased in the group with postoperative metastasis than in the non‐metastatic group (Figure 1D). Thus, high expression of lncRNA SNHG3 was positively correlated with tumour growth and metastasis in Bca tissues. Overall, survival time and disease‐free survival time analysis showed that high expression of lncRNA SNHG3 was positively related to poor clinical prognosis (Figure 1E).

### 3.2 Knockdown of lncRNA SNHG3 suppressed the growth, metastasis and EMT of bladder cancer cells in vitro

**FIGURE 2:**
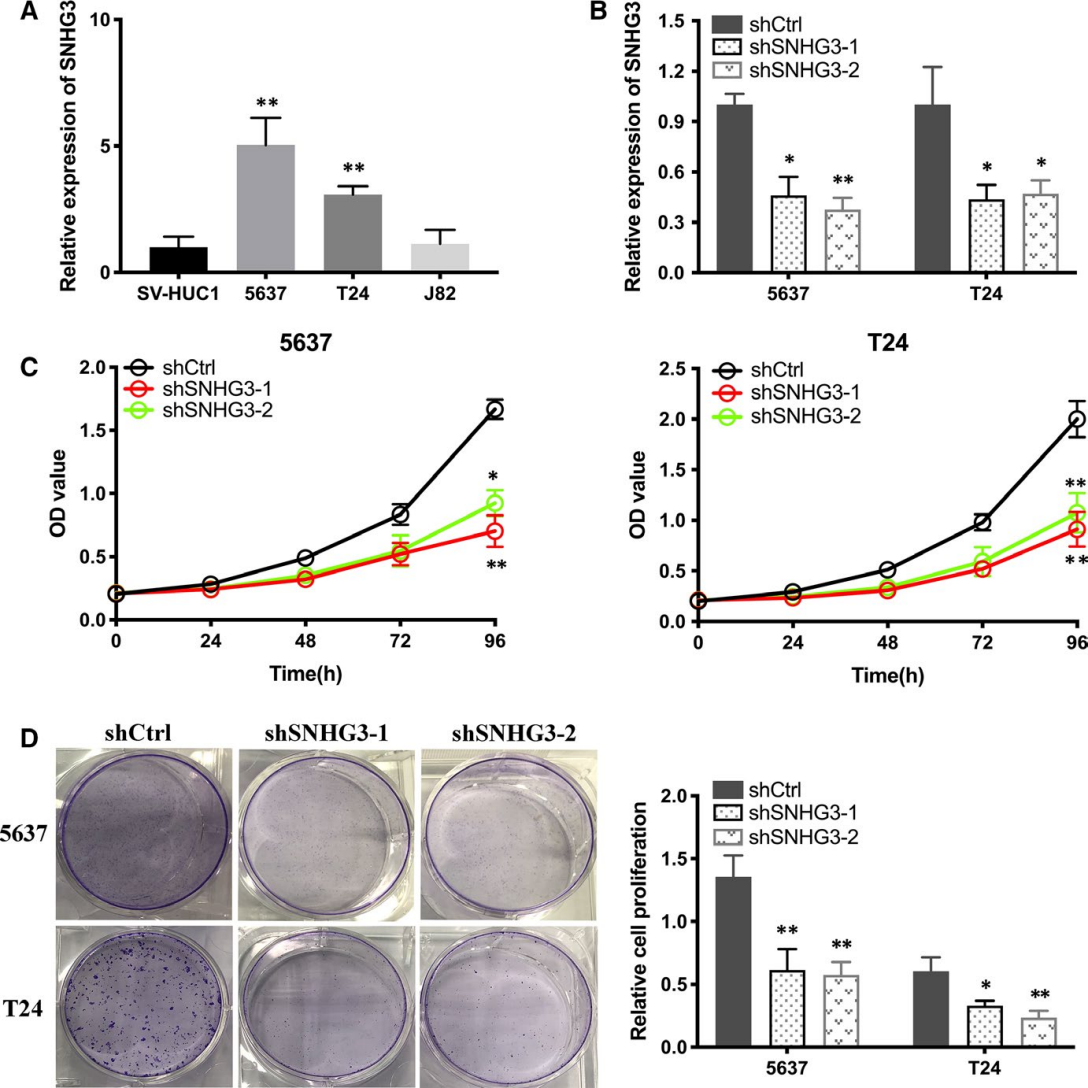
Knockdown of lncRNA SNHG3 decreased the proliferation of bladder cancer cells in vitro. A, Relative expression of lncRNA SNHG3 in several Bca cell lines compared that in normal epithelial cell line. B, The efficiency of shRNAs (shSNHG3‐1 and shSNHG3‐2) was confirmed by qRT‐PCR. (C and D) CCK‐8 assay and colony formation assay showed that knockdown of lncRNA SNHG3 decreased the proliferation of Bca cells. Data are represented as mean ± SD. *P < 0.05; **P < 0.01

qRT‐PCR assay showed that lncRNA SNHG3 expressed higher in 5637 and T24 cell lines compared to normal bladder epithelial cell line (Figure 2A). To assess whether lncRNA SNHG3 was functionally involved in Bca cells, we purchased shRNAs targeting SNHG3 (shSNHG3‐1 and shSNHG3‐2) and transfected them into 5637 and T24 cells, respectively. qRT‐PCR was performed to confirm the shRNAs were both effective (Figure 2B). CCK‐8 assay showed that knockdown of lncRNA SNHG3 decreased the proliferation of Bca cells in vitro (Figure 2C). Similarly, colony formation assay revealed that inhibited lncRNA SNHG3 reduced the proliferation of Bca cells (Figure 2D).

**FIGURE 3:**
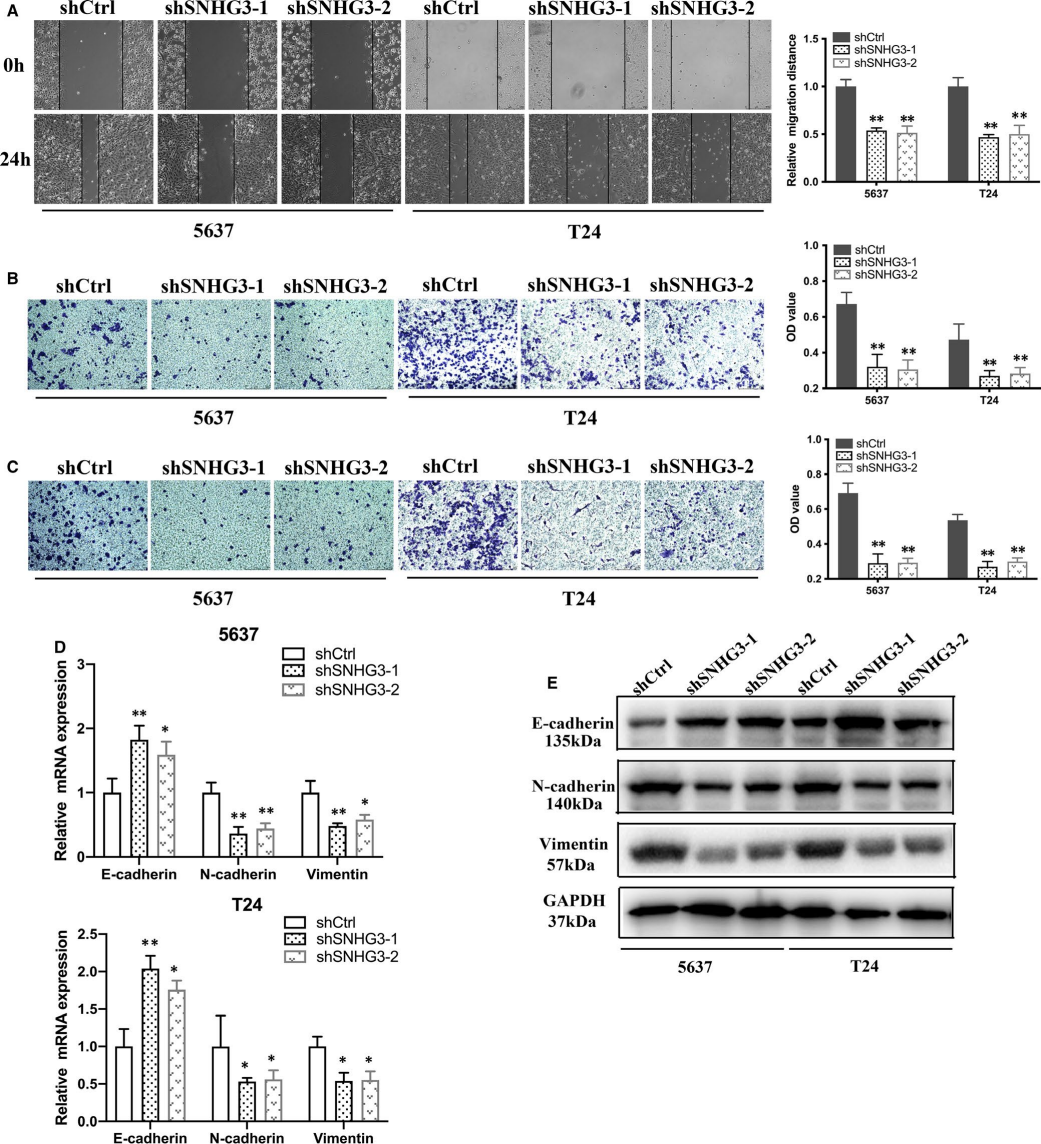
Knockdown of lncRNA SNHG3 reduced migration, invasion and EMT of bladder cancer cells in vitro. A, Wound healing assay indicated that knockdown of lncRNA SNHG3 reduced the migratory ability of Bca cells. (B and C) Transwell migration and invasion assay indicated that knockdown of lncRNA SNHG3 reduced the invasive activity of Bca cells. (D and E) mRNA expression level of EMT markers were detected by qRT‐PCR and Western blotting assay, respectively. Knockdown of lncRNA SNHG3 increased E‐cadherin expression and decreased N‐cadherin and vimentin expression in Bca cells. Data are represented as mean ± SD. *P < 0.05; **P < 0.01

As shown in wound healing assay, knockdown of lncRNA SNHG3 suppressed the migratory ability of Bca cells (Figure 3A). Transwell migration and invasion assay indicated that transfection of shlncRNA SNHG3 inhibited the invasive activity of Bca cells (Figure 3B,C). The mRNA expression level of EMT markers was detected by qRT‐PCR. It is obvious that knockdown of lncRNA SNHG3 inhibited N‐cadherin and vimentin expression, yet enhanced E‐cadherin expression in Bca cells (Figure 3D). Western blotting assay showed the similar results and confirmed the changes in expression of EMT markers at protein level (Figure 3E). Therefore, lncRNA SNHG3 functionally promoted the malignant phenotype of bladder cancer cells in vitro.

### 3.3 LncRNA SNHG3 predominantly distributed in cytoplasm and acted as a sponge for miRNA‐515‐5p

**FIGURE 4:**
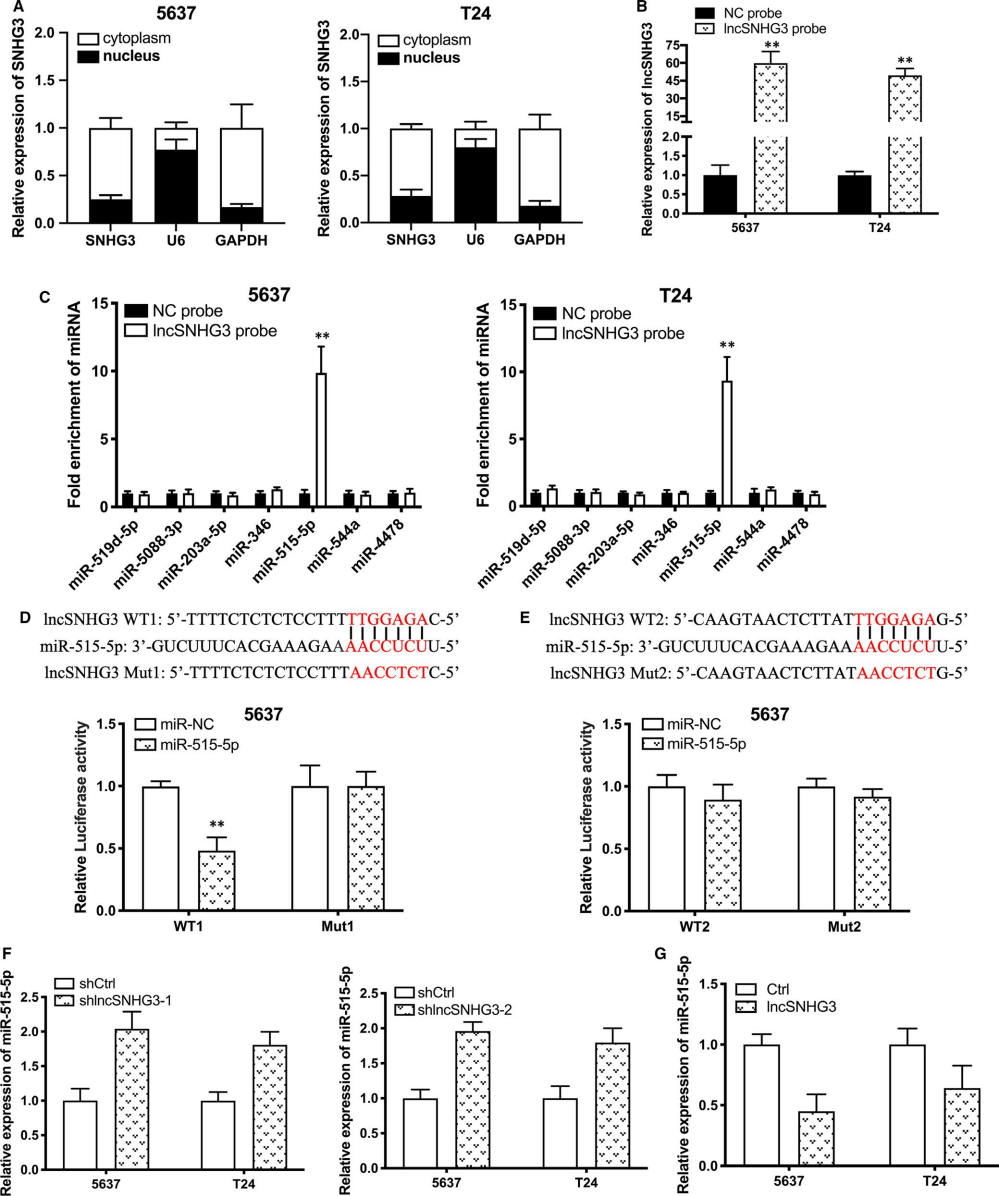
lncRNA SNHG3 directly interacted with miRNA‐515‐5p. A, Nuclear/cytoplasmic fractionation assay revealed that lncRNA SNHG3 primarily distributed in cytoplasm of Bca cells. B, The efficiency of lncRNA SNHG3 probe was verified by qRT‐PCR. C, RNA pull‐down assay showed the significant enrichment of miR‐515‐5p from lncRNA SNHG3 probe. (D and E) Bioinformatics analysis predicted that two probable sequences of lncRNA SNHG3(WT1 and WT2) are complementary to the seed sequence of miR‐515‐5p. Dual‐luciferase assay showed that co‐transfection of lncRNA SNHG3 WT1 and miR‐515‐5p significantly inhibited luciferase activity (D), whereas co‐transfection of lncRNA SNHG3 WT2 and miR‐515‐5p acted conversely (E). F, Expression of miR‐515‐5p in the Bca cells, which were transfected with shlncRNA SNHG3, was detected by qRT‐PCR. G, Expression of miR‐515‐5p in the Bca cells, which were transfected with pcDNA‐3.1 lncRNA SNHG3, was detected by qRT‐PCR. Data are represented as mean ± SD. *P < 0.05; **P < 0.01

To further explore the mechanism how lncRNA SNHG3 modulates tumorigenesis, we used nuclear/cytoplasmic fractionation assay. The experimental results showed that lncRNA SNHG3 was primarily distributed in cytoplasm of Bca cells (Figure 4A). miRDB database (http://mirdb.org/miRDB/) predicted that miR‐519d‐5p, miR‐5088‐3p, miR‐203a‐5p, miR‐346, miR‐515‐5p, miR‐544a and miR‐4478 have putative interactive sites with lncRNA SNHG3. To assess which microRNA was directly interacted by lncRNA SNHG3, we firstly performed qRT‐PCR to verify the efficiency of lncRNA SNHG3 probe (Figure 4B). Secondly, the enrichment on the biotin‐labelled probe applied for RNA pull‐down assay was examined by qRT‐PCR. According to the significant enrichment of miR‐515‐5p compared to control group, the analysis suggested that lncRNA SNHG3 directly interacted with miR‐515‐5p in bladder cells (Figure 4C). Thirdly, the two potential sequences of lncRNA SNHG3(WT1 and WT2) that are predicted complementary to the seed sequence of miR‐515‐5p were mutated (Mut1 and Mut2) and used to construct dual‐luciferase reporter vector. Dual‐luciferase reporter assay showed that co‐transfection of lncRNA SNHG3‐WT1 and miR‐515‐5p significantly inhibited luciferase activity than control group. However, this inhibition was not observed in lncRNA SNHG3‐WT2 nor lncRNA SNHG3‐Mut group (Figure 4C,D). Thus, WT1 is the core binding site for lncRNA SNHG3 to sponge miR‐515‐5p under an ceRNA manner. Additionally, qRT‐PCR determined that miR‐515‐5p expression increased or decreased due to suppression or overexpression of lncRNA SNHG3 in Bca cells (Figure 4F,G). It demonstrated the ceRNA mechanism from another angle.

### 3.4 Inhibition of miR‐515‐5p reversed silencing lncRNA SNHG3‐induced suppression of malignant phenotype suppression in Bca cells

**FIGURE 5:**
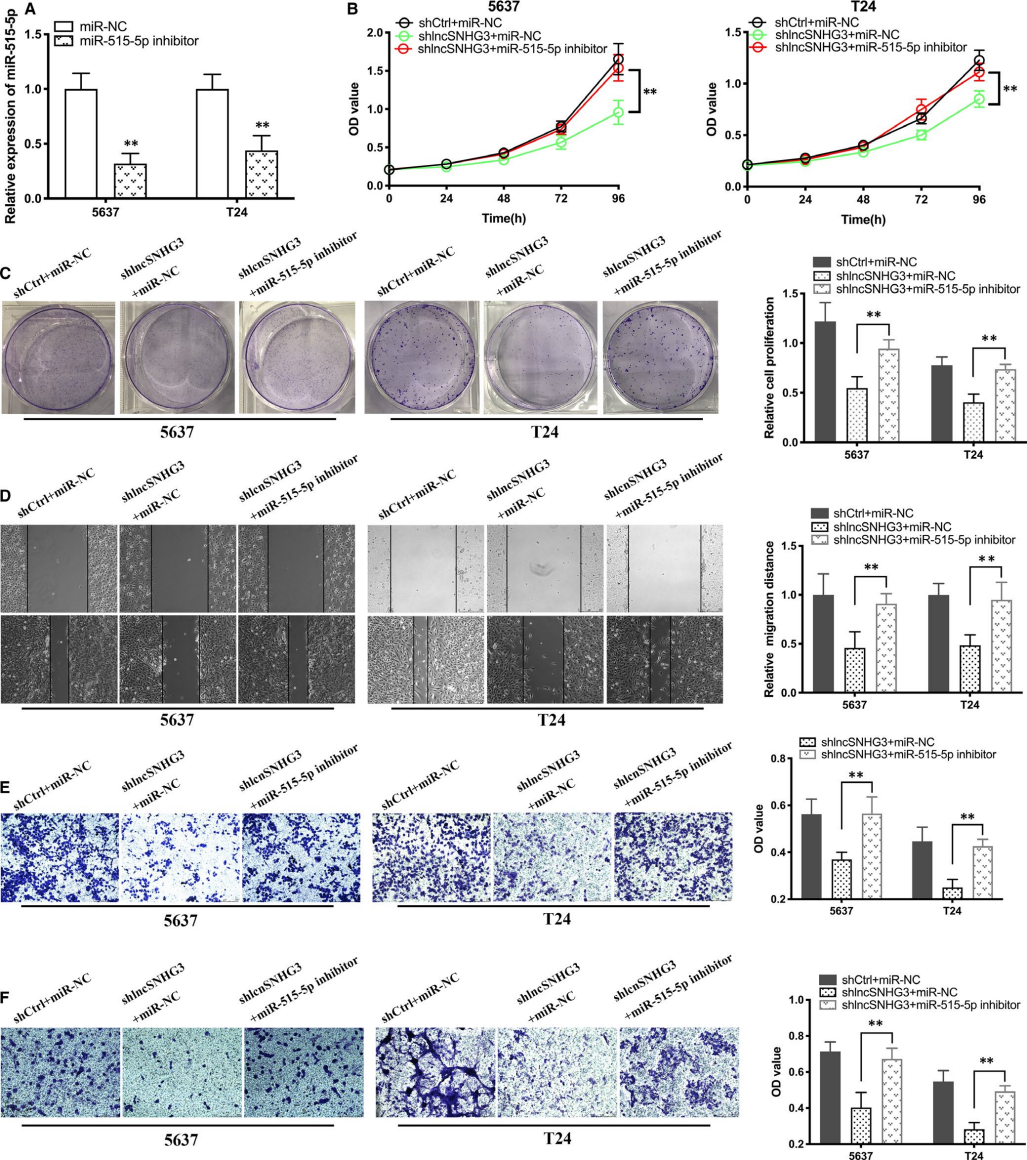
Inhibition of miR‐515‐5p reversed silencing lncRNA SNHG3‐induced suppression of malignant phenotype suppression in Bca cells. A, The effect of miR‐515‐5p inhibitor was tested and verified by qRT‐PCR. (B and C) Inhibition of miR‐515‐5p reversed the decreasing proliferative capacity induced by shlncRNA SNHG3‐1. (D and E) Inhibition of miR‐515‐5p reversed the decreasing migratory ability induced by silenced lncRNA SNHG3. F, Transfection of miR‐515‐5p inhibitor reversed the decreasing invasive ability induced by silenced lncRNA SNHG3. Data are represented as mean ± SD. *P < 0.05; **P < 0.01

We explored whether lncRNA SNHG3 targeting miR‐515‐5p to promote the malignant phenotypes in Bca cells. Firstly, the efficiency of miR‐515‐5p inhibitor was tested and verified by qRT‐PCR (Figure 5A). As CCK‐8 assay and colony formation assay showed inhibition of miR‐515‐5p significantly reversed silencing lncRNA SNHG3‐induced suppressed proliferative activity of Bca cells (Figure 5B,C). Wound healing assay showed that transfection of inhibited miR‐515‐5p significantly reversed silencing lncRNA SNHG3‐induced migration decreasing of Bca cells (Figure 5D). Transwell migration and invasion assay revealed that inhibition of miR‐515‐5p reversed silencing lncRNA SNHG3‐induced invasive suppression of Bca cells (Figure 5E,F). Above results showed that miR‐515‐5p was involved in the mechanism underlying the functions of lncRNA SNHG3 in vitro.

### 3.5 LncRNA SNHG3 positively modulated GINS2 expression via sponging miR‐515‐5p

**FIGURE 6:**
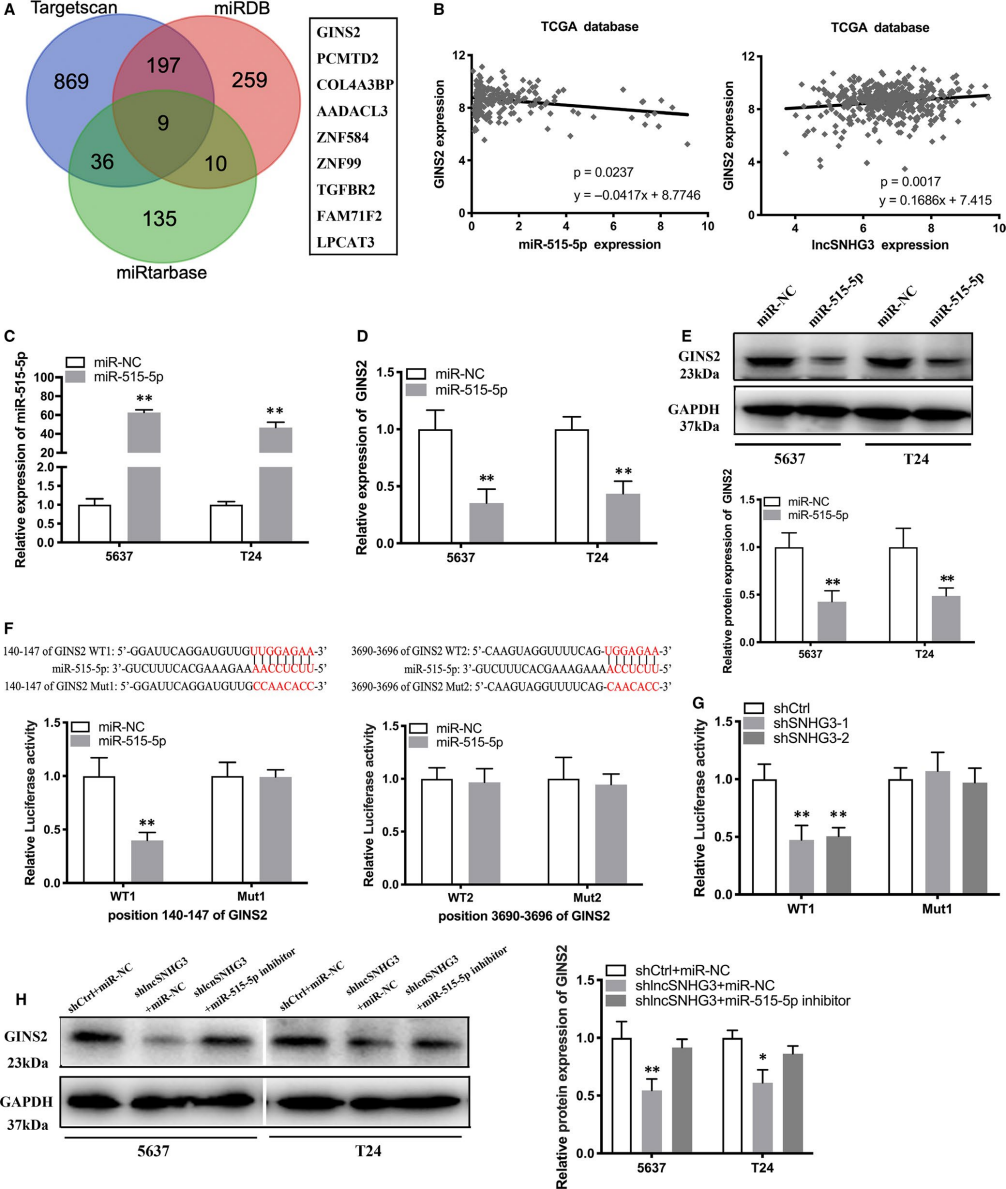
lncRNA SNHG3 positively modulated GINS2 expression via sponging miR‐515‐5p. A, Original data obtained from bioinformatics databases predicted 9 candidate genes targeted by miR‐515‐5p. B, Statistical correlation analysis showed that miR‐515‐5p expression was negatively correlated with GINS2 expression in Bca tissues, whereas lncRNA SNHG3 expression was positively correlated with GINS2 expression in Bca tissues. C, Efficiency of miR‐515‐5p mimic was confirmed by qRT‐PCR. (D and E) qRT‐PCR and Western blotting assay demonstrated that miR‐515‐5p expression levels were negatively correlated with GINS2 expression levels in Bca cells. F, Bioinformatics analysis predicted two probable 3’UTR sequences of GINS2 (WT1 and WT2) complemented to the seed sequence of miR‐515‐5p. Dual‐luciferase reporter assay showed that co‐transfection of WT1 and miR‐515‐5p significantly inhibited luciferase activity. G, Dual‐luciferase reporter assay determined that co‐transfection of WT1 and shlncRNA SNHG3 significantly inhibited luciferase activity. H, Reduced miR‐515‐5p reversed GINS2 expression inhibition induced by shlncRNA SNHG3 in Bca cells. Data are represented as mean ± SD. *P < 0.05; **P < 0.01

To identify the potential genes of miR‐136‐5p, we conducted bioinformatics analysis by using the TargetScan, miRDB, and miRTarBase databases. We found 9 candidate genes were potential targets for miR‐515‐5p (Figure 6A). We investigated the correlation between several genes and miR‐515‐5p by using TCGA database. Statistical correlation analysis determined that expression of miR‐515‐5p was correlated negatively with expression of GINS2, whereas expression of lncRNA SNHG3 was positively correlated with expression of GINS2 in Bca tissues (Figure 6B). Furthermore, we confirmed miR‐515‐5p mimic (Figure 6C) and found that expression of miR‐515‐5p was correlated negatively with expression of GINS2 in Bca cells (Figure 6D). Besides, Western blotting assay verified the negative correlation between miR‐515‐5p and GINS2 at protein level in Bca cells (Figure 6E). Bioinformatics analysis predicted two binding sites on 3’UTR sequences of GINS2 (WT1 and WT2) complemented to the sequence of miR‐515‐5p. Therefore, we performed dual‐luciferase reporter assay and confirmed the effective binding sites on the 3’UTR sequence of GINS2 for miR‐515‐5p was WT1 (Figure 6F). Subsequently, we performed another dual‐luciferase reporter assay, which determined that lncRNA SNHG3 positively regulated GINS2 expression though the above valid binding sites (WT1) (Figure 6G). Finally, Western blotting assay was taken out to confirm the above results at protein level. Transfection of miR‐515‐5p inhibitor increased GINS2 expression reduced by shlncRNA SNHG3‐1 at protein level in Bca cells (Figure 6H). Current research suggested that lncRNA SNHG3 functioned though a miR‐515‐5p/GINS2 axis in vitro.

### 3.6 Knockdown of lncRNA SNHG3 inhibited the tumour growth and EMT of Bca in vivo

**FIGURE 7:**
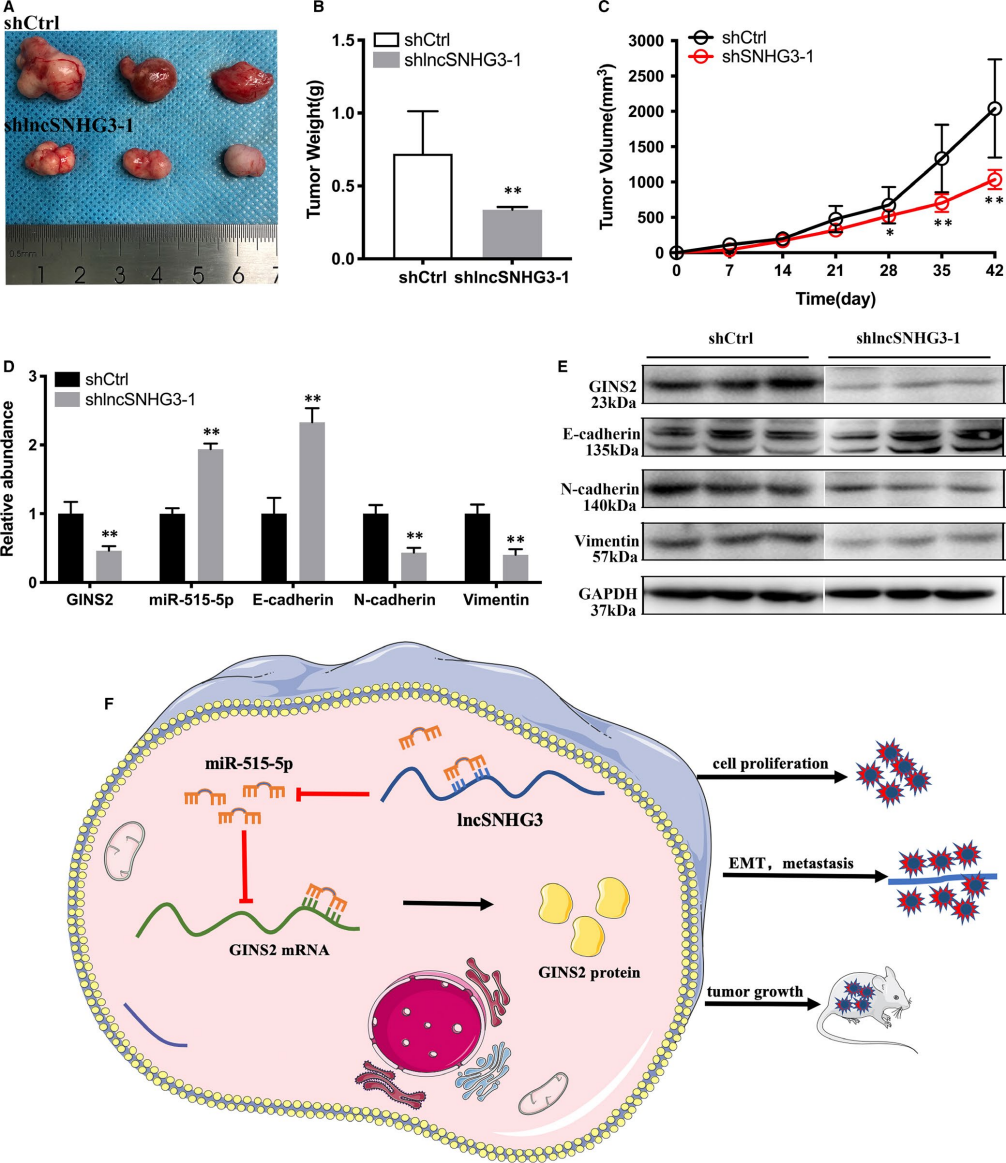
Knockdown of lncRNA SNHG3 suppressed progression of Bca cells in vivo. A, Tumours collected from two groups of nude mice. B, Tumours weighted significantly lighter in shlncRNA SNHG3‐1 group than that in control group. C, Tumours of two groups were measured and noted every week. D, Expression of GINS2, miR‐515‐5p and EMT markers was detected by qRT‐PCR. E, Western blotting assay showed that knockdown of lncRNA SNHG3 decreased N‐cadherin and vimentin expression, yet increased E‐cadherin expression in vivo. F, Schematic illustration of lncRNA SNHG3/miR‐515‐5p/ GINS2 axis. Data are represented as mean ± SD. *P < 0.05; **P < 0.01

To confirm the role of lncRNA SNHG3 in bladder cancer progression in vivo, we generated 5637 cells transfected stably with silenced lncRNA SNHG3‐1 or empty vector and injected into two groups, respectively. After 6 weeks, tumours removed from the group with down‐regulation of lncRNA SNHG3 obviously showed less size and weight than control group (Figure 7A,B). As recorded weekly, mice in the shlncRNA SNHG3 group had the smaller volume than shCtrl group (Figure 7C).

We used qRT‐PCR to detect expression of GINS2, miR‐515‐5p and EMT markers in operated tumour tissues. We found that knockdown of lncRNA SNHG3 inhibited expression of GINS2, N‐cadherin and vimentin, and up‐regulated expression of miR‐515‐5p and E‐cadherin in contrast (Figure 7D). We also performed western blotting assay to detect expression of GINS2 and EMT markers in vivo. It turned out that knockdown of lncRNA SNHG3 inhibited GINS2, N‐cadherin and vimentin expression, and up‐regulated E‐cadherin expression of bladder cancer in vivo (Figure 7E). As shown in Figure 7F, lncRNA SNHG3 positively regulated GINS2 expression through targeting miR‐515‐5p, hence promoted malignant phenotypes of Bca cells both in vitro and vivo.

## 4 DISCUSSION

Growing evidence has suggested that long non‐coding RNAs (lncRNAs) play a key role in the morbidity and progression of cancer.^23^ LncRNA SNHG3 was identified as an oncogene in the development of various cancers, including hepatocellular carcinoma, colorectal cancer and lung cancer.^17^, ^18^, ^19^ However, the role of lncRNA SNHG3 in bladder cancer remains unknown. In our study, we first reported that lncRNA SNHG3 was significantly up‐regulated in Bca tissues and positively related to the larger tumour size and postoperative metastasis. Then, we found that knockdown of lncRNA SNHG3 significantly suppressed proliferation, migration, invasion and EMT of Bca cells in vitro.

EMT is orchestrated by a complicated and multi‐factorial network, including regulators of different signalling pathways.^24^ Previous studies have suggested that EMT is correlated with neoplastic invasion and progression in various malignant tumours.^25^, ^26^ Interesting, lncRNAs have been reported participating in metastasis processes such as EMT in multiple cancer.^27^, ^28^, ^29^ As reported recently, T‐cell leukaemia 1 upstream neural differentiation‐associated RNA (TUNAR) acted as a promoter of EMT and lead to generation of stem cell characteristics, and promoting progression and metastasis in BC.^30^ LncRNA ES1 (LINC01108) and lncRNA‐Hh increased the stem cell characteristics of bladder cancer.^31^, ^32^ In our study, we discovered that knockdown of lncRNA SNHG3 repressed the EMT process of Bca.

Accumulating evidence suggests that lncRNAs perform important functions in regulating genes that influence tumour proliferation, apoptosis and migration, and expand our understanding of the biological behaviour of cancers, including bladder cancer.^33^, ^34^, ^35^, ^36^, ^37^, ^38^, ^39^ Notably, massive articles have pointed that lncRNAs regulate miRNAs through a ‘miRNA sponge’ mechanism as a competing endogenous RNA (ceRNA).^40^, ^41^ This mechanism also functions during cancer progression.^42^, ^43^ For instance, HOTAIR significantly influenced tumorigenesis in breast cancer by targeting miR‐20a‐5p.^44^ Similarly, NEAT1 targeted miR‐448 to enhance ZEB1 expression in bladder cancer.^45^ MALAT1 promoted TNBC progression though targeting miR‐1 and miR‐129‐5p.^46^, ^47^ By performing experiments in vitro and vivo, we revealed that lncRNA SNHG3 acted as a microRNA sponge targeting miR‐515‐5p.

miR‐515‐5p belongs to miR‐515 family along with miR‐518f, miR‐519c‐3p and miR‐519e‐5p.^48^ Interestingly, miR‐515‐5p has been pointed to act as a suppressor in several cancers such as breast cancer and lung cancer.^49^, ^50^ In another research, miR‐515‐5p was involved in an ceRNA mechanism with LINC00673 in breast cancer.^51^ Conversely, the biological function of miR‐515‐5p in bladder cancer has never been discussed. Our study suggested that miR‐515‐5p played a vital role that declined in Bca and sponged by lncRNA SNHG3.

Go‐Ichi‐Ni‐San (GINS) is a cyclic complex that regulates the cell cycle and plays a crucial role in cell apoptosis and proliferation.^52^ It is consisted of four subunits, including PSF1 (GINS1), PSF2 (GINS2), PSF3 (GINS3) and SLD5 (GINS4).^53^ GINS2 was revealed to be up‐regulated in leukaemia, breast cancer and cervical cancer.^54^, ^55^, ^56^ Yet, the role of GINS2 in the development of bladder cancer remains mysterious. By bioinformatics databases, we revealed that GINS2 is the direct target of miR‐515‐5p. miR‐515‐5p could lower GINS2 expression by binding to 3’UTR of GINS2. Subsequently, we revealed that lncRNA SNHG3 enhances GINS2 expression via sponging miR‐515‐5p, thereby enhances the tumorigenesis and EMT in bladder cancer. Even though our research uncovered the mechanism by which lncRNA SNHG3 functions as an up‐regulator in bladder cancer, there are unlimited feasibilities underlying. It is a common sense that one lncRNA may act as a sponge for more than one miRNA, therefore exerting different biological functions in cancer. Whether lncRNA SNHG3 can function as an ceRNA for other miRNAs or proteins requires further exploration.

In conclusion, our study identified that lncRNA SNHG3 acted as an oncogene in bladder cancer. Additionally, suppression of miR‐515‐5p reversed silencing lncRNA SNHG3‐induced suppression of malignant phenotype in bladder cancer cells. Furthermore, we revealed that SNHG3 escalated GINS2 expression by sponging miR‐515‐5p under a ceRNA mechanism. Therefore, our study indicated that lncRNA SNHG3 may serve as a novel diagnostic and therapeutic target of bladder cancer.

## CONFLICT OF INTERESTS

The authors declared no competing interests.

## AUTHOR CONTRIBUTION

**Guangcheng Dai:** Methodology (equal); Writing‐original draft (equal). **Chenchen Huang:** Data curation (equal); Methodology (equal); Writing‐original draft (equal); Writing‐review & editing (equal). **Jinhui Yang:** Writing‐original draft (equal); Writing‐review & editing (equal). **Lu Jin:** Formal analysis (supporting); Software (supporting). **Kai Fu:** Investigation (supporting); Validation (supporting). **Feng Yuan:** Visualization (equal); Writing‐review & editing (equal). **Jin Zhu:** Conceptualization (supporting); Data curation (supporting). **Boxin Xue:** Funding acquisition (lead); Supervision (lead).

## ETHICAL APPROVAL

This study has been approved by the ethics committee institution of Soochow University. Every Bca patients included in this study were informed and signed consents.

## Supporting information

Table S1‐S2

## Data Availability

The data sets supporting the conclusions of this manuscript are included within the manuscript.
