# Supplementary material for: LncRNA SNHG3 promotes bladder cancer proliferation and metastasis through miR‐515‐5p/GINS2 axis
Source: J Cell Mol Med. 2020 Jun 28;24(16):9231–43. doi: 10.1111/jcmm.15564 (PMC7417716; doi:10.1111/jcmm.15564)
Supplement: Supplementary file 1 — Table S1‐S2 [file JCMM-24-9231-s001.docx]

**Supplementary Table S1**. Primers used for Real time PCR

| **Target gene** | **Primer (5’-3’)** |
| --- | --- |
| SNHG3 | F: GACTTCCGGGCACTTCGTAA |
|  | R: TGCTCCAAGTCTGCCAAAGA |
| GINS2 | F: CGTCCAGCATCATGCAATGG |
|  | R: CACATGCGCATTCTCAGAGC |
| GAPDH | F: AATGGGCAGCCGTTAGGAAA |
|  | R: GCGCCCAATACGACCAAATC |
| U6 | F: TGCGGGTGCTCGCTTCGGCAGC |
|  | R: CCAGTGCAGGGTCCGAGGT |

Abbreviations: F, forward; R, reverse; GAPDH, glyceraldehyde 3‐phosphate dehydrogenase; PCR, polymerase chain reaction.

**Supplementary Table S2**. Primers used for Real time PCR of microRNAs

| **Target miRNA** | **Primer (5’-3’)** |
| --- | --- |
| miR-515-5p | CAGTTCTCCAAAAGAAAGCAC |
| [miR-519d-5p](http://mirdb.org/cgi-bin/mature_mir.cgi?name=hsa-miR-519d-5p) | CAGCCTCCAAAGGGAAG |
| [miR-5088-3p](http://mirdb.org/cgi-bin/mature_mir.cgi?name=hsa-miR-5088-3p) | GCAGTCCCTTCTTCCTG  R: CACATGCGCATTCTCAGAGC |
| [miR-203a-5p](http://mirdb.org/cgi-bin/mature_mir.cgi?name=hsa-miR-203a-5p) | GAGTGGTTCTTAACAGTTCAACA |
| [miR-544a](http://mirdb.org/cgi-bin/mature_mir.cgi?name=hsa-miR-544a) | GCAGATTCTGCATTTTTAGCAAG |
| [miR-4478](http://mirdb.org/cgi-bin/mature_mir.cgi?name=hsa-miR-4478) | GCAGGAGGCTGAGCTG |
